# MRI Visualization and Distribution Patterns of Foreign Modeling Agents: A Brief Pictorial Review for Clinicians

**DOI:** 10.1155/2021/2838246

**Published:** 2021-11-29

**Authors:** Leslie-Marisol Gonzalez-Hermosillo, Victor-Hugo Ramos-Pacheco, Daisy-Carolina Gonzalez-Hermosillo, Alicia-Maria-del-Consuelo Cervantes-Sanchez, Alejandro-Eduardo Vega-Gutierrez, Sergey K. Ternovoy, Ernesto Roldan-Valadez

**Affiliations:** ^1^Escuela de Medicina, Universidad de Guadalajara, Jalisco State, Mexico; ^2^Department of Magnetic Resonance, Hospital General de Mexico “Dr Eduardo Liceaga”, 06720 Mexico City, Mexico; ^3^Department of Pathology, Hospital General de Mexico “Dr Eduardo Liceaga”, 06720 Mexico City, Mexico; ^4^I.M. Sechenov First Moscow State Medical University (Sechenov University), Department of Radiology, 119992 Moscow, Russia; ^5^A.L. Myasnikov Research Institute of Clinical Cardiology of National Medical Research Center of Cardiology of the Ministry of Health of Russia, 127005 Moscow, Russia; ^6^Hospital General de Mexico “Dr Eduardo Liceaga”, 06720 Mexico City, Mexico

## Abstract

Since the ancient Egyptians, people have always been worried about their physical appearance. Nowadays, for some cultures like Latin American, physical appearance depends on the context, and the concept of beauty is to have wider hips and more prominent buttocks. One way to achieve these goals is to inject foreign modelants that include some oils to modify certain body regions. Until today, the search continues to find a modelling agent that is nonteratogenic, noncarcinogenic, and not susceptible to infection and can stay at the spot where it was injected (not migration). This review is aimed at providing a brief, comprehensive assessment of the use of modeling agents and summarizes some key imaging features of filler-related complications. The topics of this review are historical data, epidemiology, classification of dermal fillers (xenografts, hyaluronic acid derivatives, autografts, homografts, synthetic materials), adverse reactions, imaging method used in the detection of injectable fillers, MRI patterns observed in complications of injectable fillers, and histological findings of immune response, treatment, and conclusions. We present several classifications of injectable fillers based on composition, degradation, and complications. Additionally, readers will find some representative cases of the most common locations of injectable fillers demonstrating their infiltrative MRI patterns.

## 1. Introduction

People have always been worried about their physical appearance. They strive to improve this, putting through their bodies numerous ways since the ancient Egyptians used animal oils, salt, alabaster, and sour milk to improve their skin aesthetically. Nowadays, study results show that characteristics that make an individual attractive include clarity, symmetry, harmony, and vivid color [1]. In a Latin American context, the concept of beauty is to have wider hips and more prominent buttocks [2]. Injectable methods have advantages over more traditional surgical techniques, resulting in costs related to patients and other difficulties that may significantly reduce compared with procedures performed in the operating room [3].

To get better results in the future, we have to understand the facts through the history that have failed, and so, try not to repeat the same mistakes; the common aim through time is the desire to make the suitable material to replace volumes and fill lines in the face. What has been looked for is a modeling agent that is nonteratogenic, noncarcinogenic, and not susceptible to infection and can stay at the spot where it was injected (not migration) [4].

This review is aimed at providing a brief, comprehensive assessment of the use of modeling agents and summarizes some key imaging features of filler-related complications. We present several classifications of injectable fillers based on composition, degradation, and complications. Additionally, readers will find some representative cases of the most common locations of injectable fillers demonstrating their infiltrative MRI patterns.

### 1.1. Historical Data

The first report on using a foreign substance for “cosmetic” purposes dates back to 1899 [2]. A physician named Gersuny used paraffin for testicular prostheses in a patient who underwent bilateral orchiectomy for testicular tuberculosis [2, 5]. Almost when paraffin started to spread worldwide, reports on delayed reactions to these substances began to appear. *Paraffinoma* was used to describe the granulomatous foreign-body reaction resulting from paraffin injection [2, 4].

During the late 1800s, autologous fat injections were used for facial augmentation. In 1893, Neuber was the first to use autologous fat for soft tissue augmentation [3]. Autologous fat started being popular with the use of high vacuum suction with blunt cannulas in 1982. Since then, different techniques have evolved for fat harvest and transfer to the face, most of the time with inconsistent results [4].

Another autograft described in the literature was Isolagen, in the eagerness to find a superior injectable product for dermal augmentation, which led to the development of this agent in the mid-1990s [3].

The application of other injectable agents such as beeswax, lanolin, and mineral oils had undesirable results. So, in the 1960s, liquid silicone started being famous as a cosmetic treatment. It was first used in Japan during the 1940s for breast augmentation. Over the following years, it was observed that this agent would migrate and fistulize, which developed the term “siliconoma” to describe the granuloma in the injection site [3, 4].  Table 1 shows some of the historical terms used for the description of modelling agents.

RSNA: Radiological Society of North America; MRI: magnetic resonance imaging.

Not until 1972, Miyoshi adopt the term “modelants agent disease” in patients with breast prostheses [5]. Currently, the modeling agent disease has become a severe public health problem to shape certain body regions.

### 1.2. Epidemiology

Juarez Duarte et al. observed in his Latin America-study about MRI patterns of modeling agents in the gluteal region that range of ages varied as follows: 20-29 years, 6.6% of the patients; 30-39 years, 43% of the patients; 40-49 years, 36.6% of the patients, and 50-59 years, 13.3% of the patients. The type of substances registered was to 21 patients, mineral oil (70%); to 3 patients, liquid silicone (10%); to 3 patients, guaiacol (10%); to 2 patients, vegetable oil; and to 1 patient, automobile oil (3%) as referred for the patients [5].

Almost 60 years ago, in 1962, dermal fillers counted as 2.2 million women had received silicone breast implants in the USA and Canada [9].

In 1994 (almost thirty years ago), researchers from the department of rheumatology of Harvard Medical School published about the immune reactions associated with the injection of silicone, paraffin, and petroleum jelly [9].

### 1.3. Classification of Dermal Fillers

**Figure 1:**
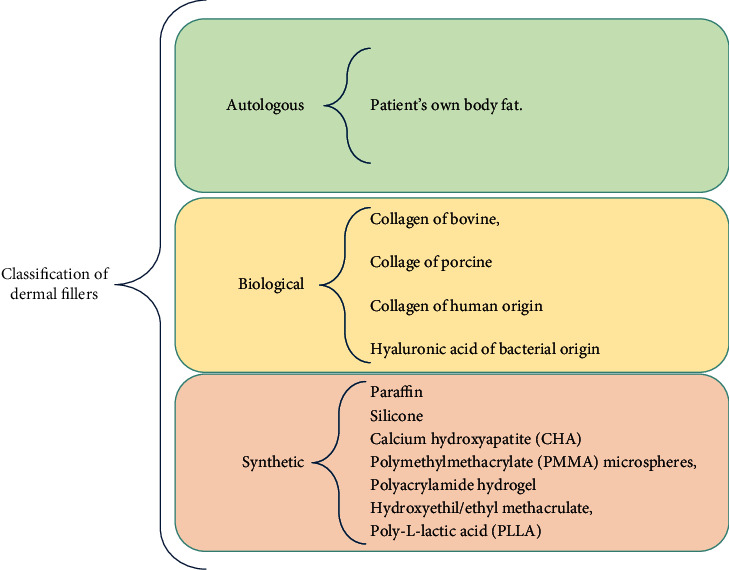
Classification of the dermal fillers according to their nature: autologous, biological, and synthetic [10].

Dermal fillers brought to the esthetic field a significant advance. As shown in Figure 1, its classification can be divided into three types, depending on their nature: autologous, biological, and synthetic [10].

**Figure 2:**
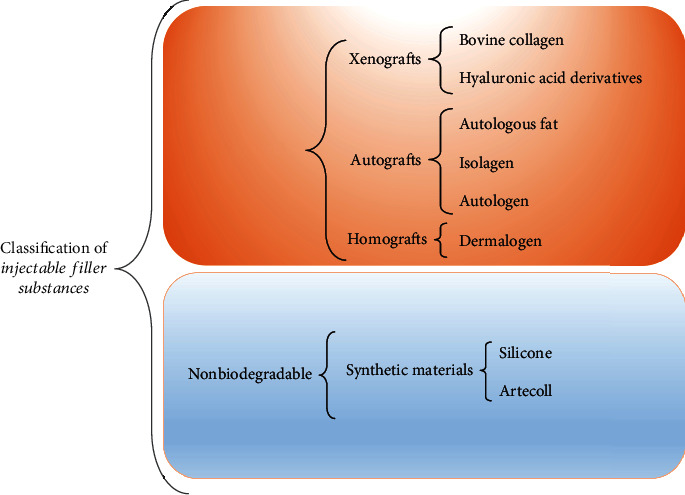
Classification of the injectable filler substances according to their biodegradability [3].

Injectable filler substances may also be classified into nonbiodegradable and biodegradable products. These two categories can be subdivided into xenografts, autografts, homografts, and synthetic products, Figure 2 [3, 11].

#### 1.3.1. Xenografts

Bovine collagen is the most widely used dermal filler. Three bovine collagen products are commercially available: Zyderm-I, Zyderm-II, and Zyplast [3].

Zyderm-I was approved for the FDA in 1981; because of significant resorption of the material over time, and a second formulation called Zyderm-II was approved for the FDA in 1983. Zyderm-II is identical to Zyderm-I, just for the fact that it contains a higher collagen concentration [3].

Zyplast, which was accepted for the FDA in 1985, contains collagen with glutaraldehyde processing, making the material less susceptible to enzymatic degradation and less immunogenic. Zyderm-I and Zyderm-II retain aprox. 30 and 60%, respectively, whereas Zyplast retains nearly 100% of its original injected volume [3, 6].

#### 1.3.2. Hyaluronic Acid Derivatives

Hyaluronic acid (HA) belongs to the family of glycosaminoglycans. These molecules are found in the extracellular matrix (ECM) of connective tissues. Their main characteristic is to attract water into the ECM, conferring more turgor. Hyaluronic acid has the unique property of being identical in all species. The FDA has approved no derivatives from hyaluronic acid in the United States [3].

Hylaform is the commercially available form derived from processed rooster comb. It is contraindicated in patients with a history of allergies to avian products. In 2001, it was introduced two more forms of Hylaform: the Hylaform Fineline (for superficial rhytids) and the Hylaform Plus (for deeper furrows) [3]. Besides these two forms of HA, in December of 2003, it was approved for the FDA Restylane, a hyaluronic acid product. This approval was a boom in the popularity of injectable fillers [6].

#### 1.3.3. Autografts

Autologous fat: over the years, its popularity has waned. It has a high rate of graft resorption and degree of volume loss. Fat grafting has the disadvantage of requiring a donor site [3].

Isolagen: isolagen contains the patient's living cells, autologous dermal fibroblasts. The live fibroblasts are injected into the mid-dermis for soft tissue augmentation; 2-4 treatments are necessary to accomplish the desired correction [3].

Autologen consists of the dermal extracellular matrix that has been isolated from a patient's skin; it allows the isolation of matrix components like collagen (type I, III and VI), elastin, fibronectin, and glycosaminoglycans. It requires multiple treatments to accomplish the desired result [3].

#### 1.3.4. Homografts

*(1) Dermalogen*. It is very similar to Autologen, except that the dermal matrix is derived from human cadavers from accredited tissue banks. It is probably necessary for a single skin test that shows no reaction for three days to rule out adverse reactions [3].

#### 1.3.5. Synthetic Materials

Silicone: the basic structure of the current silicone implants is a silicone elastomer device, and its content is a combination of low and high molecular weight polydimethylsiloxane (PDMS) monomers [12, 13]. The silicone mimics the shape of normal tissue, creating a “natural” feeling augmentation. In 1991, the FDA declared the use of injectable silicone illegal, but it is still used in other countries [3]. One theory with great acceptance about the effect of silicone on the body is the development of autoimmune disease in genetically predisposed patients [13].

Artecoll is a combination of both synthetic and biological components. It contains polymethylmethacrylate (PMMA), bovine collagen, and lidocaine. PMMA prevents phagocytosis by macrophages. It is indicated for deeper wrinkles, nasolabial folds, and lip augmentation. Over 100,000 patients have been treated with Artecoll in Europe and Canada since 1994 [3].

### 1.4. Adverse Reactions

Bovine collagen has a high potential effect on allergic reactions. For this reason, intradermal skin testing is mandatory. The local manifestations reported are abscess formation, tissue necrosis, and granulomatous foreign body reactions at the injection site. Systemic reactions are rare and have reported headaches, nausea, arthralgias, rash, or anaphylactoid reactions [3, 6].

In the case of hyaluronic acid derivatives, immunogenic reactions are infrequent [3, 6]. However, a cutaneous hypersensitivity was reported in one patient after the third injection into the nasolabial folds. In three patients, a delayed inflammatory reaction was observed at the site of injection [3].

Fat grafting has no risks of allergic reactions and bio incompatibility because of its autologous nature [3].

The use of isolagen, namely autologous cells, avoids biocompatibility, immune rejection, allergic reactions, and infections transmission. At present, no adverse reactions have been reported [3].

Autologen has many advantages associated with autologous transplants: nonallergic, nontoxic, and nonimmunogenic. Some of the major disadvantages are the lack of available skin in patients not contemplating elective skin excision procedures and the delay in the process [3].

Dermalogen and cymetra: no allergic reactions have been observed; however, some local side effects could present, such as erythema, burning sensation, and acneiform eruptions [3].

Silicone: when this is administered in large volumes, it led to some local and systemic effects. Generally, the inflammatory reaction which is around the injected silicone is self-limited.

Local adverse reactions are chronic inflammation, migration, extrusion, ulceration, and granuloma formation. Systemic reactions such as granulomatous hepatitis, pulmonary embolism, and silicone pneumonitis have led to organ failures and deaths [3].

Artecoll: one allergic reaction has been reported, and two patients experienced hypertrophic scarring, necessitating removal of the material. Artecoll is not yet approved by FDA [3].

### 1.5. Imaging Method Used in the Detection of Injectable Fillers

One of the most significant problems physicians can face treating this disease is the amount of the agent used ignored by the patient [5].

The imaging studies are very significant in the diagnosis of foreign modeling agent reaction (FMAR). Magnetic resonance imaging (MRI) is the imaging study of choice that provides the most valuable information due to its accurate soft tissue discrimination capability. When MRI is not an option, high-frequency ultrasound (US) may evaluate inflammation and estimate the amount of modelling agent injected. The main findings in the US are increased density and echogenicity of the subcutaneous tissue [2, 10].

### 1.6. MRI Patterns Observed in Complications of Injectable Fillers

The most frequently affected areas are the buttocks. Other frequently affected regions are the breasts, lower extremities, and the face [2]. On the face incudes the perioral area, periocular region, nasolabial folds, malar fat pad, marionette lines, glabella, and lips [10].

**Figure 3:**
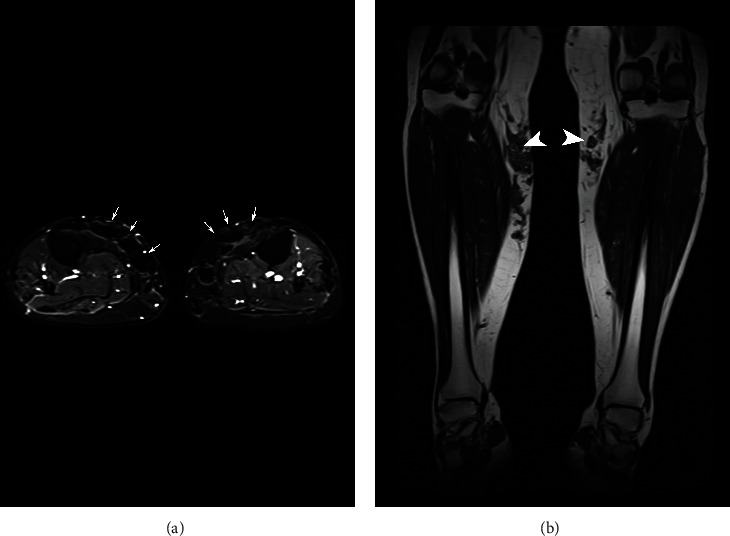
Magnetic resonance imaging using STIR (short tau inversion recovery, also known as short-TI inversion recovery) sequence. STIR is a fat suppression technique with an inversion time TI = ln(2) · T1fat, where the fat signal is zero; this equates to approximately 140 ms at 1.5 T. STIR is used to distinguish two tissue components when their T1 values are different. It allows homogeneous and global fat suppression and can be used with low-field-strength magnets. However, this technique is not specific for fat; thus, the signal intensity of tissue with a long T1 and tissue with a short T1 may cause ambiguity. In this figure, STIR shows in two planes the infiltration by the modeling agent to the legs, an unknown substance, 800 ml. (a) Axial plane: the presence of modeling material with a hypointense globular pattern that affects the anteromedial surfaces of the subcutaneous cell tissue of both legs is observed (white arrows). (b) Coronal T1 sequence: the signal of the modeling material is isointense to the muscle (white arrowheads).

Several infiltration patterns can be presented, namely, mixed, globular, linear, or pseudonodular. Many of these patterns were present in this review; as shown in Figure 3, the modeling agent infiltration (unknown what type) to the legs is with a predominant globular pattern.

**Figure 4:**
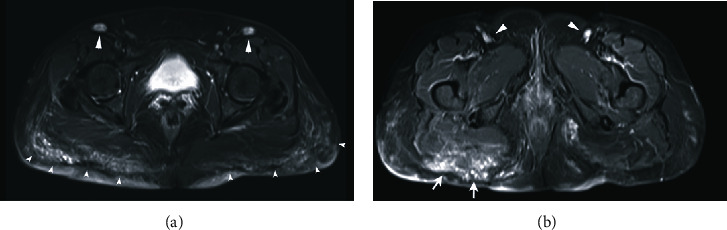
MRI using STIR sequence in the axial plane shows infiltration of the ganglia and the different patterns present in the gluteal region. Unknown substance, 2000 ml. (a) Depicts a mixed globular and diffuse pattern that affects the gluteus maximus muscle and the subcutaneous cellular tissue of the gluteal regions (small and large, white arrowheads) migration to the left ischiorectal fossa. (b) Infiltration by modelling material of the inguinal ganglia and gluteus maximus muscle (white arrows and arrowheads). Infiltration of ganglia points to the engulfment of injectable filler by macrophages and their eventual migration to regional ganglia.

In other cases, it is possible to find a mixed globular and diffuse pattern that affects the gluteus maximus muscles (Figure 4).

**Figure 5:**
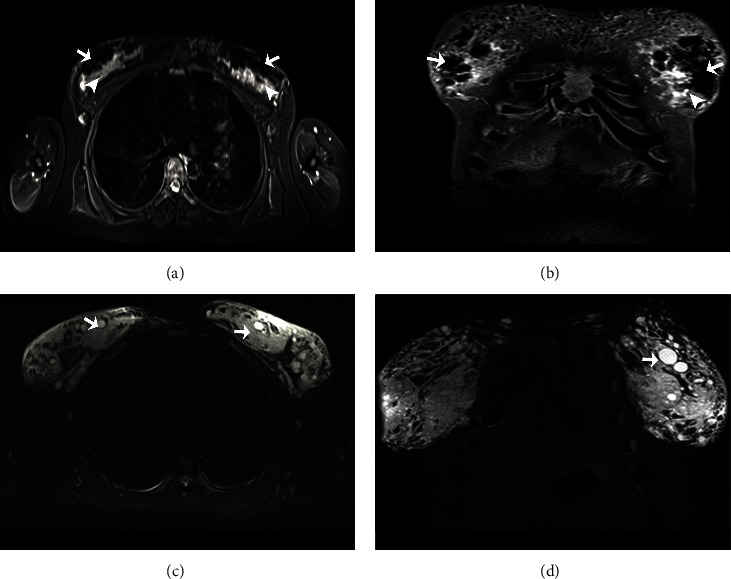
Magnetic resonance imaging STIR sequence showing different patterns in the breasts, in two patients, with the infiltration of silicone and other substances. Patient 1: it was used silicone, an approximate amount of 1250 ml. (a) Axial plane: there is infiltration by modelling material with a mixed and nodular pattern, with hypointense signal in the superficial portion (white arrows) and a diffuse pattern with hyperintense signal in the deep part of both breasts (white arrowheads); right axillary ganglion with modeling infiltration is also identified. (b) Coronal plane: the hypointense signal's globular pattern also affects the intermammary region's subcutaneous cellular tissue (white arrows). Patient 2: it was a mixture of mineral oil, car's oil, and silicone, with an approximate amount of 2000 ml. (c) Axial plane: a globular pattern of hyperintense signal is observed (white arrows), associated with diffuse hyperintensity changes due to diffuse infiltration in both breasts. (d) Coronal plane: globular pattern of hyperintense signal predominantly in the left breast (white arrow).

For the breast, mixed and nodular patterns have been reported in the same patients; in other cases, an isolated globular pattern infiltrating both breasts has been identified (Figure 5).

**Figure 6:**
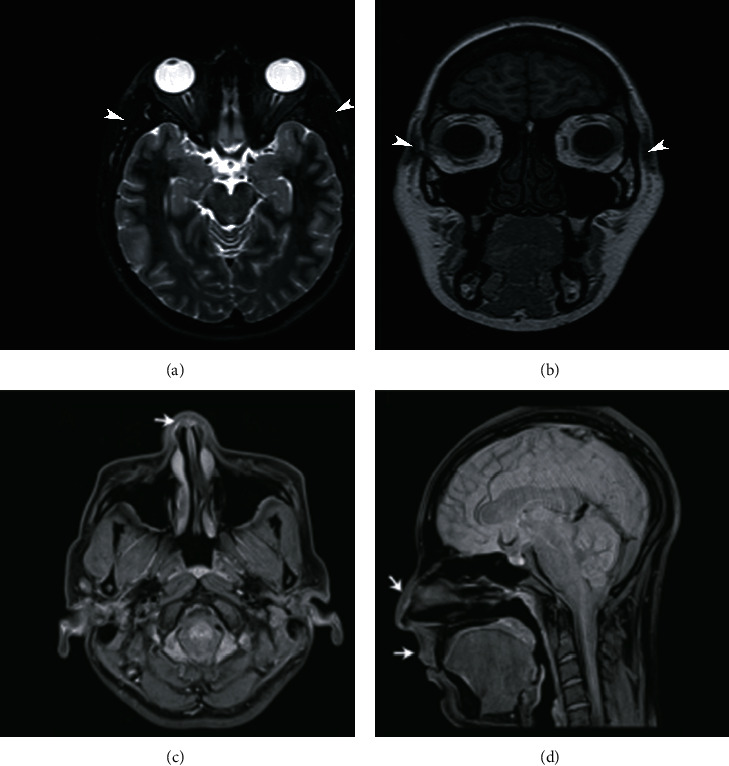
FatSat proton density sequence magnetic resonance was showing complications in the facial area in two different patients. Patient 1: this patient used polymethacrylate, an amount of 30 ml. (a) Axial T2 sequence: infiltration with a diffuse pattern of hyperintense signal is observed in the bilateral periorbital subcutaneous cellular tissue (white arrowheads). (b) Coronal T1 sequence, the signal in this sequence is hypointense, visualizing bilateral periorbital inflammation (white arrowheads). Patient 2: he used hyaluronic acid as a modeling agent with an approximate amount of 25 ml. (c) Axial plane: diffusely increased signal intensity is observed in the superficial soft tissues of the nasal pyramid due to infiltration of modelling material (white arrow). (d) Sagittal plane: in addition to infiltration with a diffuse pattern in the nasal pyramid, there is infiltration in the lips (white arrows). The importance of mentioning the amount of modelling agent and injected region is that these patients usually do not undergo surgery on the face (this case was from a patient with one of the most considerable amounts injected). For these patients, surgeons keep the clinical decision expectant; patients are managed with vigilance to avoid postsurgical aesthetic complications such as scars. Readers should remember that the face region usually receives smaller injected amounts than other areas), therefore, do not develop autoimmune systemic inflammatory syndromes as often as other regions.

In the face, diffuse patterns have been found in the periorbital and the nasal pyramid regions; infiltration into the lips has been reported (Figure 6).

Abscess, cellulitis, noninflammatory nodules, and granulomas are the most common modelling agent-related complications so that imaging can be helpful in the differential diagnosis. Most commonly, the depth of the affectation reaches down to the muscles [2, 10].

Silicone has significant MRI features such as calcium hydroxyapatite calcifications, whereas other agents have overlapping imaging features [10]. Most fillers like hyaluronic acid, and collagen, have intensity patterns compatible with high water content.  Table 2 presents the main imaging patterns of dermal fillers observed in computed tomography (CT) and MRI.

#### 1.6.1. Clinical Manifestations

The age of presentation can vary widely. The historical data of each patient depends on the nature and amount of the injected substance [2]. One significant disadvantage of using most injectable fillers is resorption over time, provoking repeated applications to achieve the desired results. Moreover, it is common to experience transient erythema, edema, ecchymosis, and induration for the first 72 hours after injection. These findings alone are not indicative of allergic reactions. Persistent local symptoms, including pruritus, suggest hypersensitivity reactions to the injectate [3, 11].

The most common local findings are inflammation (edema, increased temperature), induration (from panniculitis-like to severe wood-hard fibrosis), scars (atrophic and hypertrophic), discoloration (hypo and hyperpigmentation), necrosis, ulcer, and exposure to the injected material [2, 18].

Systemic manifestations depend on the amount and nature of the injected material; patients can present fever, malaise, and unrelated or related to infection, as well as arthralgia, myalgia, and Raynaud's phenomenon. Systemic granulomatous reactions are associated with FMAR [2]; Table 3 shows injectable fillers' local and systemic manifestations [2, 3, 11].

#### 1.6.2. Complications

It is essential to mention that it is very likely that patients develop complications; specifically, a subset of patients develop defined autoimmune diseases such as systemic lupus erythematosus (SLE), rheumatoid arthritis (RA), Systemic Sclerosis (SSc), overlap syndrome, (autoimmune hemolytic anaemia) AHA, ulcerative colitis, and thyroiditis make it harder for the patients to recover faster [18].

Some patients may present with nonspecific manifestations of autoimmune rheumatology disease (ARD), and others can be presented with manifestations of ARD (SLE, rheumatologic arthritis, SSc). Human adjuvant disease (HAD) is associated with exposure to foreign substances that can act as adjuvants to develop rheumatic manifestations [18].

Female patients have reported feeling worse during the menstrual period. In male patients, worsening is associated with the use of hormonal steroid injection [2].  Table 4 presents the main MRI findings in dermal filler-related complications.

Short-term complications are commonly associated with the procedure itself and the reaction host response to the injected material. These complications occur within days or weeks.

Long-term complications are associated with the delayed host response [10]; Table 5 shows the short- and long-term complications related to the use of injectable fillers.

**Figure 7:**
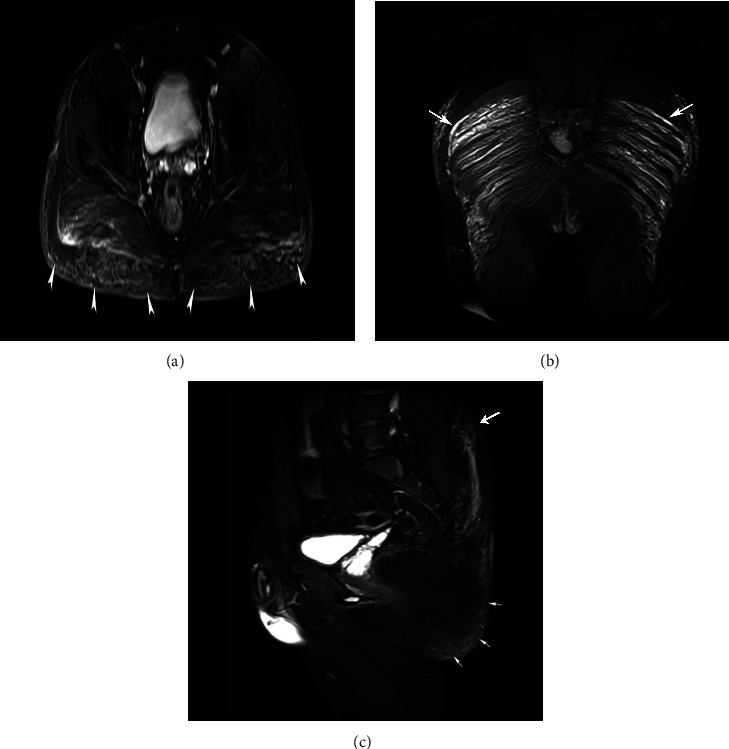
STIR sequence magnetic resonance shows three planes to describe the infiltration and migration of the modeling agent to the ischiorectal fossa. The substance used by this patient was biopolymers and an approximate amount of 1000 ml. (a) Axial plane: infiltration by modeling material with a diffuse pattern that infiltrates the gluteus maximus muscles (white arrowheads) with migration towards the ischiorectal fossa. (b) Coronal plane: the affection by modeling material is presented in the subcutaneous cellular tissue (white arrows). (c) Sagittal plane: identifying migration of the modeling material towards the lumbar region (long white arrow).

An example of a long-term complication can be observed in Figure 7, which depicts through MRI the migration of the modeling agent from the gluteus maximus muscles towards the ischiorectal fossa and the lumbar region. The substance used by this patient was biopolymers and an approximate amount of 1000 ml.

**Figure 8:**
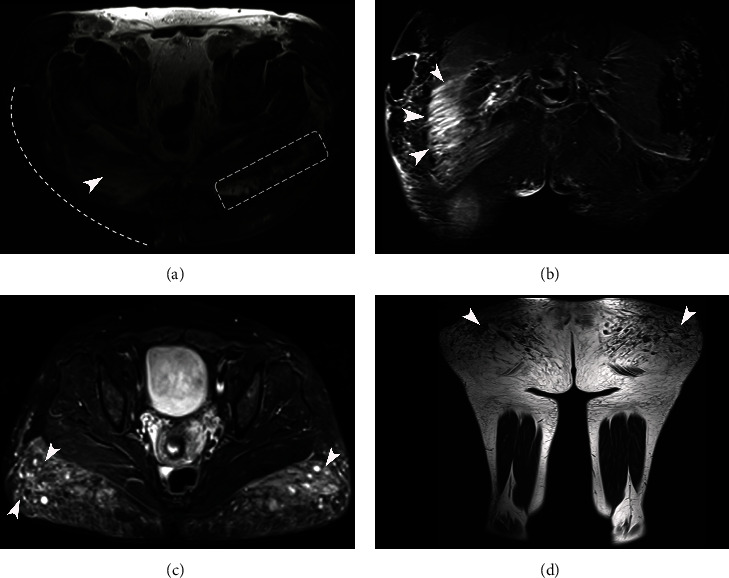
Magnetic resonance imaging STIR sequence applied in two different patients with complications in the gluteal region. Patient 1: (a) T2 axial sequence: the modeling material's presence affects all the subcutaneous cellular tissue of the hips with a diffuse pattern left gluteal region. Note the hypointense signal in this sequence, with inflammatory changes of the major gluteal muscles (dotted white rectangle); the patient presented resection of the subcutaneous cellular tissue of the right gluteal region due to a complication of necrosis (dotted white line) and can be observed the infiltration to the right gluteal muscle (white arrowhead). (b) Coronal sequence: the modeling material migration to the ischiorectal fossae (white arrowheads). Patient 2: unknown substance, an approximate amount of 1000 ml. (c) Axial plane shows infiltration with modeling material with a mixed globular and diffuse pattern that only affects the subcutaneous cellular tissue of the gluteal regions (white arrowheads). (d) Coronal plane: the signal of the modeling material is hypointense (white arrowheads).

The complication of necrosis is emphasized in two patients in Figure 8; patient 1 had resection of the subcutaneous cellular tissue of the right gluteal region due to necrosis; she used two substances, mineral oil and silicone, counting an amount of 3000 ml.

### 1.7. Histological Findings of Immune Response

**Figure 9:**
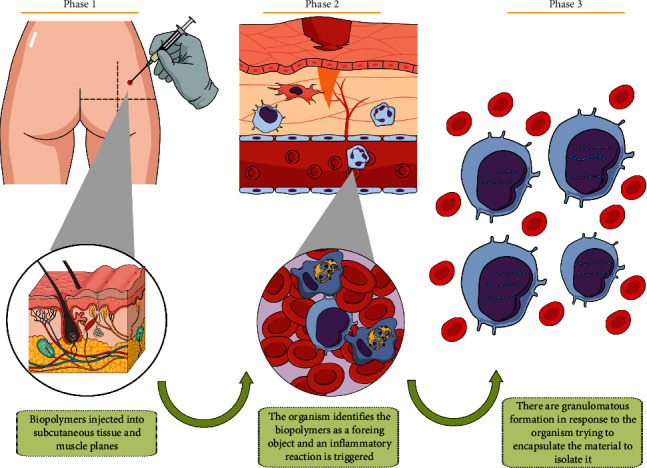
The three steps of the granulomatous formation.

The main feature in histology is chronic granulomatous inflammation, leading to capsule formation, where there are foamy histiocytes or multinucleated giant cells with phagocytised foreign material. Because FMAR is deeply injected, both the subcutaneous fat and the dermis are commonly affected [13]. The three steps of the granulomatous formations are depicted in Figure 9.

**Figure 10:**
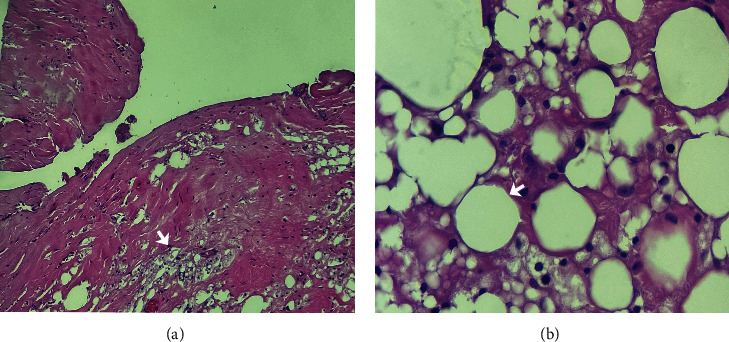
Histological findings in a tissue sample with dermal fillers.

Histological sections show scant mammary parenchyma extensively substituted by dense collagen on the internal surface, partial coverage by synoviocytes (Figure 10(a)). Other findings include multiple areas representing deposits of exogenous material with the formation of irregular optically clear vacuoles that vary in size and shape among the adipose tissue and within the macrophages below the subcapsular synovial membrane (Figure 10(b)).

### 1.8. Treatment

In mild cases, proper wound care could be the only necessary treatment, but surgical procedures and systematic therapy may be helpful in more severe cases.

Local treatment may be presented with incision and drainage of the abscess, surgical debridement with infiltrated areas, and reconstructive procedures; the foreign material must be removed whenever possible [2, 18]. For example, a case of penile paraffinoma showed improvement after topical application of potassium permanganate soaks [2].

Systemic treatment like steroids and immunosuppression may be necessary when systemic inflammation is still present. There is an initial administration of deflazacort during the first 30 to 45 days. If no response is recorded, the addition of azathioprine, colchicine, thalidomide, hydroxychloroquine, or mycophenolate should be considered for three months. After three months, if the disease is still active, it could be switch to cyclophosphamide or etanercept [2]. When we are talking about HAD, the treatment depends on the clinical and serological predominant manifestations.

Most patients will develop chronic relapses, but this will depend on the injection type and amount of modeling agent [2].

## 2. Conclusions

In conclusion, there has been reported worldwide that nearly all fillers can produce adverse events, and the classification used in this pictorial review (biodegradable and nonbiodegradable) cannot be made for the type of adverse reactions.

More and more people are currently interested in using modeling agents, which persists in different socioeconomic strata, being more incident in developing countries, despite the prohibitions addressed by the FDA.

There has to be special attention to the adverse events related to these products, especially nonbiodegradable agents, because there are more challenging to treat. Clinicians should rely on MRI findings given the efficiency of this diagnostic modality in examining soft tissues; advanced MRI modalities like spectroscopy can supplement the evaluation of the effects of modeling.

## Figures and Tables

**Table 1:** Historically used terms for a description of modeling agents.

Name	Date of publication in the medical literature	Journal/institution	Imaging method	References
Autologous fat	The late 1800s	Facial plastic surgery	—	[4]
Modeling agent: paraffin	The late 1900s	Facial plastic surgery.	—	[4]
Silicone (Asia's syndrome)	The 1940s	Facial plastic surgery	—	[4, 6]
Hyaluronic acid fillers	2001	Facial plastic surgery	—	[3]
Biopolymers	2020	RSNA	MRI	[7]
Dermal fillers	2020	RSNA	MRI	[7]
Iatrogenic allogenosis	2020	RSNA	MRI	[8]

**Table 2:** Description of the imaging patterns of dermal fillers presented in the CT and MRI [10, 14–17].

Dermal fillers	Imaging patterns
Autologous fat fillers	The filler has the appearance of low attenuation soft tissue using CT. On MRI, it appears as a thin pseudocapsule [10].

Collagen fillers	On CT, collagen appears as fluid attenuation, and the infiltered subcutaneous fat shows a streaky appearance. MRI may show hypointense on T1 W images and hypertense on T2 W and STIR images because of its high water content [10, 14].

Calcium hydroxyapatite (CHA) fillers	On CT, CHA presents as intense defined linear streaks or masses that are rounded. After 12 months, the CT filler density diminishes because microspheres get absorbed, and after 24 months, the filler can disappear. On MRI, T1 W, and T2 W images, CHA fillers exhibit a low to intermediate signal intensity [10, 17].

Hyaluronic acid (HA) fillers	On CT, soft tissue attenuation is a common sign of HA fillers. On MRI, HA fillers show substantially hyperintense on T2 W and STIR sequences and hypointense on T1 W sequences due to their high water content. In the first six months after injection, minor postcontrast enhancement is visible, indicating increasing vascularization of the injected tissue [10, 15].

Poly-l-lactic acid (PLLA)	On CT, it shows soft-tissue attenuation. On MRI, T2 W images appear hypointense.

Polyalkylimide and polyacrylamide hydrogels (PAAG)	On MRI, PAAG fillers show as hyperintense on T2 W and hypointense on T1 W sequences, and there is no evidence of postcontrast enhancement [10, 16].

Silicone oil filler	On MRI, its appearance varies depending on its viscosity and purity. On T1 W images, the low viscosity silicone oil appears somewhat hyperintense to water, iso- or slightly hypointense on T2 W images, and hyperintense on the “silicone only” sequence. On T2 W images, high viscosity silicone oil is hypointense. All tissues except silicone are suppressed in a “silicone-only” sequence On CT, silicone seems slightly hyperdense [10, 14].
Paraffin	Calcific spherical foci and soft tissue density nodules with a calcific rim are CT findings of “paraffinoma” [10].

**Table 3:** Local and general manifestations associated with injectable fillers [2, 3, 11].

Local	Systemic
Inflammation, edema, erythema, ecchymosis Hiperpigmentation or hypopigmentation Scars Ulcerations, necrosis, sclerosis Migration of the substance Infection Fistulas	Pain, fever, malaise Joint pain, arthralgia, myalgia, and Raynaud's phenomenon. Systemic granulomatous reactions Lymphadenopathy Depression and self-esteem problems.

**Table 4:** Main MRI findings in dermal filler-related complications.

Dermal filler-related complication	Evaluation for imaging (MRI)
Abscess formation	The skin's natural barrier is interrupted by the filler injection, increasing the possibility of infection [10]. Formated as a lobulated fluid collection with the enhancement of the rim and adjacent fat stranding on MRI. The abscess can show restricted diffusion on DWI [10, 19].

Foreign body granuloma (FBG) and noninflammatory nodule (NIN)	A nonallergic chronic granulomatous reaction that develops very slowly after injection of the filler. This can develop many years after filler injection. It is most frequently seen after long-standing silicone oil infusion [10]. According to Girolamo et al., the MRI findings were that the nodular or diffuse patterns enhancement around the filler suggests FBG. In contrast, the nongranulomatous inflammation did not show enhancement and suggested NIN [10, 20].

Cellulitis	Streaky enhancement in the subcutaneous fat corresponds to cellulitis [10].

Migration of fillers and overfilling	The substances migrate through lymphatic or haematogenous routes and could mimic a malignant pathology of distant organs. Overfilling can appear as diffuse facial asymmetry or a focal lump [10].

Scarring and lymph node enlargement	MRI may depict a thick band-like subcutaneous deposition of silicone associated with diffuse soft tissue swelling and postcontrast enhancement [10].

**Table 5:** Short- and long-term complications associated with the use of injectable fillers [10].

Complications		
Short-term	Mild	Severe
	Erythema Bruising Hyperthermia Swelling Hypersensitivity Nodule formation Lumpiness in the injection area	Tissue necrosis Blindness Cerebral infarct

Long-term	Foreign body granuloma (FBG) Abscess formation Migration of filler Disfiguring nodules Tissue necrosis and ulcer Persistent discoloration Scarring	

## Data Availability

The data used to support this study's findings are available from the corresponding author upon reasonable request.
